# Controlling the Anchoring Effect through Transcranial Direct Current Stimulation (tDCS) to the Right Dorsolateral Prefrontal Cortex

**DOI:** 10.3389/fpsyg.2017.01079

**Published:** 2017-06-28

**Authors:** Jianbiao Li, Xile Yin, Dahui Li, Xiaoli Liu, Guangrong Wang, Liang Qu

**Affiliations:** ^1^Reinhard Selten Laboratory, Business School, Nankai UniversityTianjin, China; ^2^Labovitz School of Business and Economics, University of Minnesota Duluth, DuluthMN, United States; ^3^Neural Decision Laboratory, Weifang UniversityWeifang, China; ^4^MBA School, Zhejiang Gongshang UniversityHangzhou, China

**Keywords:** DLPFC, tDCS, anchoring effects, willingness to pay (WTP), selective accessibility, cognitive sets

## Abstract

Selective accessibility mechanisms indicate that anchoring effects are results of selective retrieval of working memory. Neuroimaging studies have revealed that the right dorsolateral prefrontal cortex (DLPFC) is closely related to memory retrieval and performance. However, no research has investigated the effect of changing the cortical excitability in right DLPFC on anchoring effects. Transcranial direct current stimulation (tDCS) can modulate the excitability of the human cerebral cortex, while anodal and cathodal tDCS are postulated to increase or decrease cortical activity, respectively. In this study, we used tDCS to investigate whether effects of increased or decreased right DLPFC excitability influence anchoring effects in willingness to pay (WTP) experiments. Ninety participants were first randomly assigned to receive either anodal, cathodal, or sham stimulation of 15 min, then they performed a valuation task regarding WTP. The results showed that anchoring effects were negatively related to activities of right DLPFC: the anodal stimulation diminished anchoring effects while the cathodal stimulation increased anchoring effects. These outcomes provide one of the first instances of neural evidence for the role of the right DLPFC in anchoring effects and support psychological explanations of the selective accessibility mechanisms and cognitive sets.

## Introduction

Anchoring effect, which is considered one of the most robust cognitive biases in human judgment and decision making (Furnham and Boo, 2011), describes a phenomenon that an individual’s decision tends to bias toward the initial information presented to the individual. Meanwhile, anchoring effect is still observed, even when the anchor is arbitrary and irrelevant to the judgment made by the individual (Epley and Gilovich, 2001; Furnham and Boo, 2011; Ma et al., 2015). The first evidence of anchoring effect was reported by Tversky and Kahneman (1974). In their experiment, participants were first shown a random number (i.e., the anchor) between 0 and 100 determined by spinning a wheel similar to that on Wheel of Fortune. The participants were then requested to respond to two consecutive judgment tasks that included a comparative assessment and an absolute assessment. In the comparative assessment, participants were asked to indicate whether the percentage of African countries in the United Nations was higher or lower than the anchor. In the absolute assessment, participants were instructed to give their best estimate of that percentage. Results showed that participants’ answers were strongly affected by the anchor.

Following Tversky and Kahneman (1974), researchers have designed numerous experiments to validate and advance the anchoring effect (for a review, see Furnham and Boo, 2011). In an influential study, Ariely et al. (2003) showed that anchoring effects also affected people’s economic behaviors. They found that irrelevant anchors, represented by social security numbers, were positively correlated with participants’ willingness to pay (WTP), the maximum price an individual was willing to sacrifice to get a good. This stream of experiments was considered as the standard anchoring paradigm (Mussweiler and Strack, 1999; Epley and Gilovich, 2001, 2005) in which the anchor was usually given by the experimenter or a random device such as a wheel.

The theoretical explanation for the standard anchoring paradigm is related to processes of selective accessibility of working memory (Epley and Gilovich, 2001, 2005; Furnham and Boo, 2011; Ma et al., 2015; Bahník and Strack, 2016). The selective accessibility mechanism is based on the assumption that human beings may selectively retrieve their memories that are triggered by anchors (Mussweiler and Strack, 1999; Furnham and Boo, 2011). Individuals are inclined to believe that a hypothesis is right rather than wrong, that is they follow the hypothesis-consistent testing strategy (Mussweiler and Strack, 1999). When they are given information of an anchor, they attempt to generate a target value by selectively retrieving knowledge from their memories that is consistent with the anchor, even though they know the anchor is irrelevant to the answer (Chapman and Johnson, 1999; Mussweiler and Strack, 1999). These studies indicate that the formation of the anchoring effect is related to information retrieval and processing of working memory (Mussweiler and Strack, 1999; Furnham and Boo, 2011). Some studies have offered indirect empirical evidences between anchoring effects and human beings’ capacity to hold and manipulate information in working memory (Allred et al., 2016). For example, Bergman et al. (2010) found that the actual WTP for consumer goods could be manipulated by an uninformative anchor and that this anchoring effect decreased with higher cognitive ability, while Gevins and Smith (2000) suggested that cognitive ability might be related to the activity of the brain system that supports working memory.

In addition to these behavioral experiments, a few studies have applied event-related brain potential (ERP) or electroencephalography (EEG) technologies to provide neural evidences of anchoring effects (Qu et al., 2008; Ma et al., 2015). Experimenters of an EEG experiment (Ma et al., 2015) requested participants to observe random numbers and to listen to pieces of noises. Then participants reported their willingness to accept to listen to these noises. Results showed that larger P2 and late positive potential amplitudes were elicited when a higher anchor number was drawn, indicating the anticipation of more intensive pain from subsequent noises. This provided a neural confirmation of the anchoring effect.

However, it is not clear which area of the brain is related to anchoring effects. The selective accessibility theory suggests that anchoring effects are related to information selection and retrieval from working memory (Epley and Gilovich, 2001, 2005; Furnham and Boo, 2011; Ma et al., 2015; Bahník and Strack, 2016). Therefore, it is a reasonable assumption that the brain area involving information retrieval from working memory may affect the anchoring effect.

The dorsolateral prefrontal cortex (DLPFC) plays a crucial role in working memory (Curtis and D’Esposito, 2003). DLPFC is found to be associated with the performance of working memory in some prior tDCS studies (Jeon and Han, 2012; Meiron and Lavidor, 2013) and is often activated in neuroimaging studies of memory retrieval (Fletcher et al., 1998; Henson et al., 1999; Hayes et al., 2004; Straube, 2012). Moreover, Oberauer et al. (2000) differentiated working memory by means of three main functions, which are simultaneous storage and manipulation, supervision (or executive control), and coordination. They proposed that the supervisory function was associated with the prefrontal cortex in which “working memory monitored and controlled ongoing mental operations and actions, selectively activated relevant representations, and processed and inhibited irrelevant ones” (p. 1019).

A series of functional magnetic resonance imaging (fMRI) studies suggested that the right DLPFC was important in the selection of an item from memory (Rowe et al., 2000; Rowe and Passingham, 2001). These researchers found that the selection, not the maintenance of working memory items, was associated with activation of the dorsal lateral prefrontal area 46 (the right DLPFC, MNI coordinates 42, 38, 28). Furthermore, Beer et al. (2004) highlighted that the right lateral prefrontal area was related to the ability to filter out irrelevant information (i.e., anchors) and the ability to orient to, sustain, and manipulate relevant and useful information in working memory. These evidences suggest anchoring effects may be negatively associated with activities of the right DLPFC because the selective accessibility mechanism indicates that the formation of the anchoring effect is related to information retrieval and selection of working memory (Mussweiler and Strack, 1999; Furnham and Boo, 2011).

Many studies involving cognitive sets and task sets, which reflect the pre-configuration of cognitive processes prepared for the subsequent tasks (Rowe et al., 2007; Sakai, 2008), also suggest DLPFC play an important role in the generation of anchoring effects. The anchoring effect can be seen specified cognitive sets (e.g., the process of retrieval of working memory) triggered by anchors (Mussweiler and Strack, 1999; Furnham and Boo, 2011), while DLPFC is founded to be closely related to the maintenance of a specified set (MacDonald et al., 2000; Sakai, 2008), and switching or shifting to the appropriate cognitive set (Ravizza and Carter, 2008).

In this research, we aim to apply the transcranial direct current stimulation (tDCS) technique to analyze the neural process of anchoring effects by investigating the role of the right DLPFC. Unlike correlational methods (e.g., fMRI), tDCS provides causal evidence that a brain region is involved in a behavior of interest (Filmer et al., 2014; Riva et al., 2014; Sanchez et al., 2016). According to the selective accessibility mechanism (Chapman and Johnson, 1999; Mussweiler and Strack, 1999), we assume that if the anodal (cathodal) of tDCS was applied to increase (decrease) the activities of the right DLPFC, participants might be less (more) likely to retrieve irrelevant information consistent with anchors, thus anchoring effects would decrease (increase). To the extent we know, this is the first study to explore the role of DLPFC in anchoring effects by means of tDCS stimulation. The findings may be helpful for understanding the existing psychological hypothesis on anchoring effects.

## Materials and Methods

### Participants

Ninety healthy college students were recruited in the experiment (36 males and 54 females; mean age: 21.5 ranging from 18 to 25 years old). All participants were right-handed without ex-ante knowledge of tDCS or anchoring effects, and they didn’t have any history of psychiatric illness or neurological disorders. Participants were randomly assigned to three groups, sham stimulation group (*n* = 30, 10 males), cathodal tDCS group (*n* = 30, 14 males), and anodal tDCS group (*n* = 30, 12 males). The experiment was performed in accordance with the Declaration of Helsinki and was approved by the Ethics Committee of Business school of Nankai university. All participants provided written informed consent before taking part in the experimental task. Participants received an incentive of a fixed amount, which was about 7.46 United States dollars.

The data of two participants were excluded from further analysis. One participant in the cathodal group reported discomfort with stimulation, and we stopped the experiment. Another participant in the sham group did not understand experimental instructions and reported zeroes for all the answers. Overall, 88 subjects were kept in the sample.

### Transcranial Direct Current Stimulation (tDCS)

Transcranial direct current stimulation is a non-invasive technique where a constant current is passed from one electrode (the anode) to the other (the cathode) over a period of 8 to15 min (Filmer et al., 2014). Anodal tDCS increases cortical excitability and cathodal tDCS decreases cortical excitability (Civai et al., 2015). Therefore, tDCS can establish the causal relationship between stimulated brain area and cognitive functions of interest (for reviews, see Filmer et al., 2014; Sellaro et al., 2016).

In our experiment, all participants received tDCS delivered by a battery-driven stimulator (Neuro Conn, Germany). We placed the 35 cm^2^ target cathodal electrode for the cathodal group and anodal electrode for the anodal group over the right F4, according to the international EEG 10–20 electrode system. This was the most commonly used approach in tDCS studies of the right DLPFC (Tremblay et al., 2014; Ye et al., 2015; Wang et al., 2016). The other reference electrode was placed over Cz for both groups, which was consistent with the design of Meiron and Lavidor (2013) and Harty et al. (2014). Participants in the anodal or cathodal group were first received 15 min of stimulation. The stimulation current was constant at 1.5 mA intensity with 15 s of ramp up and down. Then, participants were requested to complete a valuation task on anchoring effects. The procedure was same for the sham group except that the current was stopped after the first 30 s. The 30 s of stimulation in the sham condition, which has been widely recognized in tDCS studies (Knoch et al., 2008; Riva et al., 2014; Civai et al., 2015; Ye et al., 2015), is suggested to be reliable as participants are not able to distinguish between tDCS and sham sessions (Gandiga et al., 2006). There is evidence show that it can mimic the itching sensation of the real stimulation without producing any significant neural altering effects on the cortex (Hummel et al., 2005; Willis et al., 2015).

### Task and Procedure

A standard design of an experiment used to investigate anchoring effects is to request participants to respond to a comparative assessment based on a given anchor and a subsequent open-ended question (Green et al., 1998). Most of the experimental tasks are designed based on participants’ general domain knowledge (Jacowitz and Kahneman, 1995; Strack and Mussweiler, 1997; Epley and Gilovich, 2001, 2005; Ren and Croson, 2013). These general knowledge tasks are typically conducted using specially designed knowledge questions that participants may not have naturally applied in their decision making. Therefore, the generalizability and validity of these tasks can be questioned (Furnham and Boo, 2011).

On the other hand, studies of real-world judgment tasks such as valuations and purchasing decisions have also demonstated that anchors can directly affect customers’ decisions (Mussweiler and Strack, 2000; Ariely et al., 2003; Furnham and Boo, 2011). For instance, Green et al. (1998) discovered that higher anchor values led to higher subsequent WTP for public goods. Furthermore, standard rational models of economic and consumer behaviors usually assume an individual’s preferences are stable (Fudenberg et al., 2012). However, the prevalence of anchoring effects can challenge the assumption, as preferences are not fixed if irrelevant anchors influence individuals’ economic decisions (Fudenberg et al., 2012). Following this notion, examining anchoring effects in the economic valuation tasks have important implications for behavioral economic theories.

In our experiment, participants were first randomly assigned to receive either anodal, cathodal, or sham stimulation of 15 min, then they were requested to respond to a valuation task. We adopted a double-blind procedure during the experiment task. There were two experimenters during our experiment in which one experimenter performed tDCS to participants, while another one directed the valuation task in another isolated area. Meanwhile, both the experimenter who directed the valuation task and participants were blind to the stimulation condition.

The valuation task we conducted in the experiment was similar to those conducted by Ariely et al. (2003), Bergman et al. (2010) and Fudenberg et al. (2012), except that we divided participants into three groups and applied tDCS technology before subjects participated in the tasks. Participants were first presented three types of ordinary consumer goods, a box of Ferrero Rocher chocolates, a bottle of average wine, and a novel of *One Hundred Years of Solitude*. The retail price for these three products ranged from 35 to 150 currency units in the country where this study was conducted. These items were briefly described by the experimenter without mentioning the market price. Participants were then requested to report the last two digits of their cell phone numbers. For each of these three products, participants were asked to answer whether they would purchase the product for the amount that was equivalent to their phone numbers’ last two digits. After answering Yes/No, participants stated their maximum WTP for the product. Participants understood that both a Yes/No answer and their WTP response were likely to determine the final purchase, based on the BDM incentive-compatible procedure (Becker et al., 1964).

## Results

Anchoring effect refers to the level of influence that irrelevant anchors (phone numbers) impose on individuals’ estimations (WTP). Therefore, participants who are biased by anchoring effects will report WTP closer to their phone numbers. Prior studies usually examined anchoring effects by investigating the significance of correlation or regression between WTP and phone numbers (Ariely et al., 2003; Bergman et al., 2010).

We first reported Pearson correlations between the random anchor (phone number) and WTP across the three goods in the three groups, sham, cathodal, and anodal, respectively (see **Tables 1–3**). For each of the five quintiles of the random anchor’s (phone numbers) distribution, we also presented average WTP.

**Table 1:** Average WTP sorted by quintile of the distribution of the phone number (sham group).

Quintile				Average WTP
(range)	Chocolate	Wine	Novel	for all products
1 (4–25)	59.167	78.286	36.429	57.238
2 (26–29)	61.167	117.000	32.667	70.741
3 (30–52)	61.333	66.667	30.667	55.333
4 (61–79)	80.333	109.000	48.000	79.267
5 (88–93)	84.000	73.600	41.000	66.200
Pearson correlation	0.363	–0.127	0.312	0.145
*p*-value	0.053	0.512	0.092	0.452

**Table 2:** Average WTP sorted by quintile of the distribution of the phone number (cathodal group).

Quintile				Average WTP
(range)	Chocolate	Wine	Novel	for all products
1 (3–26)	47.667	49.667	21.167	39.500
2 (29–37)	59.500	54.000	29.500	47.667
3 (38–59)	53.333	65.667	30.000	49.667
4 (60–88)	63.500	103.333	46.333	71.056
5 (90–97)	80.000	132.000	43.600	85.200
Pearson correlation	0.398	0.601	0.500	0.607
*p*-value	0.032	0.001	0.006	0.001

**Table 3:** Average WTP sorted by quintile of the distribution of the phone number (anodal group).

Quintile				Average WTP
(range)	Chocolate	Wine	Novel	for all products
1 (5–30)	73.167	104.000	45.000	74.056
2 (36–59)	130.000	120.833	41.333	97.389
3 (61–65)	74.833	72.333	28.500	58.556
4 (69–84)	103.333	121.667	55.000	93.333
5 (85–99)	102.500	126.667	45.833	91.667
Pearson correlation	0.162	0.115	0.044	0.142
*p*-value	0.394	0.546	0.817	0.453

In the sham group (**Table 1**), the correlations between the random anchor (phone number) and stated WTP ranged from -0.127 to 0.363. The correlations for two of three goods were significant at the 10% level (*p* = 0.053 for chocolate and *p* = 0.092 for novel). However, the correlation for wine was not significant (*p* = 0.512). The correlation between average WTP for all the goods and phone numbers was not significant (*p* = 0.452).

In the cathodal group (**Table 2**), the correlations were relatively higher than those in the sham group, ranging from 0.398 to 0.607. Furthermore, all the correlations were significant at the 5% level, two of which were significant at the 1% level (*p* = 0.032, 0.001 and 0.006, respectively). The correlation between average WTP for all goods and phone numbers was also significant at the 1% level (*p* = 0.001).

In the anodal group (see **Table 3**), the correlation coefficients ranged from 0.044 to 0.162 and were smaller than those in the cathodal group. Meanwhile, none of the correlations were significant (*p* = 0.394, 0.546 and 0.817, respectively). The correlation between average WTP for all goods and phone numbers was insignificant either (*p* = 0.453).

Further, we conducted paired *t*-tests to examine the difference between average WTP and phone numbers. The results were shown in **Table 4**, which suggested that significant differences were found for the sham (*p* = 0.005) and anodal group (*p* = 0.005) but no difference for the cathodal group (*p* = 0.212). These results suggested that anchoring effects in the cathodal group were more significant than those in the sham and anodal groups.

**Table 4:** Paired *t*-tests of average WTP and phone numbers.

Treatment	Average WTP	Average phone number	*t*	*p*
Sham	66.575	47.862	3.049	0.005
Cathodal	57.701	51.724	1.277	0.212
Anodal	83	59.867	3.072	0.005

When testing or controlling the effect of continuous phone number on WTP, the phone number should be left continuous and not categorized, otherwise there is a loss of power (Aiken et al., 1991). Therefore, ANCOVA (analysis of covariance) or multiple regressions rather than ANOVA are recommended (Aiken et al., 1991). We performed a one-way ANCOVA to examine the effect of tDCS stimulation treatments (anodal, cathodal and sham) on average WTP whilst controlling for phone number. There was a significant difference in the average WTP (*F*_2,82_ = 4.233, *p* = 0.018) between the tDCS conditions after controlling for phone number. *Post hoc* tests showed there was a significant difference between sham condition and anodal condition (*p* = 0.04), and anodal condition and cathodal condition (*p* = 0.002), while difference between sham condition and cathodal condition was insignificant (*p* = 0.238). Comparing the estimated marginal means showed that the average WTP in the cathodal group (mean = 58.546) was smaller than that in the sham group (mean = 67.161), and the average WTP in the anodal group (mean = 81.755) was larger than those in other two groups. The results of ANCOVA indicated the average WTP in the anodal condition was significant different from that in the sham condition.

We also conducted analyses of the impact of the phone number on stated WTP in several OLS models (**Table 5**) in which factors such as sex, major and GPA were controlled. In the sham group, the coefficient of the phone number on WTP was significant at the 5% level for novel (coefficient = 0.157, *p* = 0.048) and at the 10% level for chocolate (coefficient = 0.511, *p* = 0.054), while it was insignificant for wine. In the cathodal group, the coefficients for all three goods were significant at the 5% level (*p* = 0.018, 0.017, and 0.009, respectively), and the coefficient for the average WTP was highly significant at the 1% level (coefficient = 0.555, *p* = 0.008). In the anodal group, coefficients for all three goods were not significant. These results indicated that anchoring effects in the cathodal group were more significant and steady than those in the sham group, while anchoring effects in the anodal group were insignificant.

**Table 5:** OLS models of phone numbers on WTP in different stimulation and goods.

	Sham			Cathodal			Anodal		
WTP	Coefficient	*SE*	*p*	Coefficient	*SE*	*p*	Coefficient	*SE*	*p*
Chocolate	0.511	0.252	0.054	0.327	0.129	0.018	0.24	0.287	0.412
Wine	–0.199	0.226	0.387	0.846	0.33	0.017	0.188	0.52	0.72
Novel	0.157	0.076	0.048	0.307	0.107	0.009	0.011	0.12	0.931
Average	0.095	0.098	0.338	0.555	0.19	0.008	0.146	0.263	0.584

*The control variables included sex, major and GPA; standard errors are robust.*

Combing the results above, we suggested, (1) participants in the cathodal group were clearly affected by anchoring effects, as paired *t*-tests in **Table 4** showed no significant difference between the average WTP and phone numbers (*p* = 0.212), and the OLS model in **Table 5** showed the regression coefficients for three goods were all significant at the 5% level, and (2) participants in the sham group were partially affected by anchoring effects, as the OLS model in **Table 5** showed the coefficients of phone numbers on WTP were significant at the 5% level for one item and at the 10% level for two of three items, and (3) participants in the anodal group were not clearly affected by anchoring effects, as the OLS model in **Table 5** showed the regression coefficients for three goods were all insignificant, and *post hoc* tests of ANCOVA showed the anodal group differed significantly from both the cathodal group (*p* = 0.002) and sham group (*p* = 0.04). These results suggested that the anodal stimulation decreased anchoring effects, while the cathodal stimulation increased anchoring effects, supporting out hypotheses.

## Discussion

Although many behavioral experiments have been conducted to investigate anchoring effects, the neural mechanism of this effect is yet to be explored (Ma et al., 2015). Several studies using ERP or EEG methods provided some initial neural evidences of anchoring effects (Qu et al., 2008; Ma et al., 2015). However, due to the limited spatial resolution of the ERP technique, these studies didn’t reveal the brain area that was related to anchoring effects (Qu et al., 2008). This study found that the right DLPFC plays an important role in the processing of anchoring effects. The anchoring effect was negatively related to activities of right DLPFC. That is, when anodal of tDCS was used to increase activities of the right DLPFC, the anchoring effect significantly decreased. On the other hand, when cathodal of tDCS was used to decrease activities of this area, the anchoring effect significantly increased. The selective accessibility mechanism suggests that the formation of the anchoring effect is related to information retrieval and selection of working memory (Mussweiler and Strack, 1999; Furnham and Boo, 2011). A series of fMRI studies confirmed the role of the right DLPFC in the selection of an item from memory (Rowe et al., 2000; Rowe and Passingham, 2001). We integrated these studies and hypothesized that activities of the right DLPFC could affect anchoring effects. The experimental results in this study were consistent with the selective accessibility mechanism and the fMRI findings. Thus, we have provided some neural evidence to support the selective accessibility mechanism.

Compared to selective retrieval of working memory (Mussweiler and Strack, 1999), a more general neural explanation of anchoring effects can be provided from the perspective of cognitive sets. Considering the procedure in our experiment, participants firstly reported their anchors and thus established cognitive sets, reflecting their anticipation for the subsequent questions (MacDonald et al., 2000; Sakai, 2008). Particular pieces of knowledge triggered by anchors were accessed and stored in short-term memory during this process (Sugden et al., 2013). When participants were then requested to report WTP, the rules for responding to cognitive sets trigged by anchors became irrelevant to the current task (Ravizza and Carter, 2008), and participants were supposed to detect the conflicts between different rules (Mansouri et al., 2007), switch to the appropriate cognitive sets (Ravizza and Carter, 2008) and diminish the residual effect (e.g., information consistent with anchors in working memory) of a previous task set (Sakai, 2008). If participants failed to detect the conflicts and switch to the appropriate cognitive sets, information triggered by anchors would be then selectively retrieved in the valuation task (Sugden et al., 2013), and anchoring effects generated.

Our results were consistent with the viewpoints that DLPFC played important roles not only in the maintenance of cognitive sets (Rowe et al., 2007), but also in the switching or shifting among cognitive sets (MacDonald et al., 2000; Ravizza and Carter, 2008; Sakai, 2008). For example, the anodal stimulation decreased anchoring effects, which indicated participants with increased DLPFC excitability successfully switched to a new task set in the WTP task, and diminished the residual effect of anchoring related task sets. This neural path of anchoring effects also provides experimental predictions or neural mechanisms for similar effects involving short-term mindsets, such as framing, which indicates the preference between options is related to their description (Tversky and Kahneman, 1986). Macoveanu et al. (2016) performed a Prisoners Dilemma game which was either framed with a cooperation title or a competition title. They found a trend activation in DLPFC occurred when the competition framing cue was presented, but this activation did not leaded to framing effects in the decision phase. This finding also suggested that DLPFC both involve the establishment and switching of cognitive sets.

The valuation task we conducted in the experiment was similar with those conducted by Ariely et al. (2003), Bergman et al. (2010), Fudenberg et al. (2012), and Yoon et al. (2013). There were clear contradictions among the results of these studies. Ariely et al. (2003) and Bergman et al. (2010) reported that the correlations between social security numbers (anchors) and WTP were mostly significant at the 5% level and at the 10% level for all goods. Yoon et al. (2013) requested participants to inspect seven items of products, and found significant correlations for five goods at the 5% level and insignificant correlations for two good. However, Fudenberg et al. (2012) found no anchoring effects at all and did not provide a specific explanation for the inconsistency. Our results in the sham group were relatively moderate compared with these studies. We found significant correlations for two goods at the 10% level and insignificant correlation for one good.

Our results in the sham group were relatively consistent with results of Yoon et al. (2013), which showed that the strength of anchoring effects was related to different product items. A possible explanation, as Sugden et al. (2013) suggested, is that anchoring effects work primarily when the anchor value is framed in a plausible range of the price for the good. when participants firstly reported their anchors, particular pieces of knowledge were accessed and stored in short-term memory, and these items were then selectively retrieved in the WTP task only when the anchor value was framed as a plausible price for the good (Sugden et al., 2013). Compared with the other two products (market prices in our experiment were 35 and 91, respectively), wine was usually more expensive in china (the market price of wine in our experiment was 150). Therefore, the anchor for wine might be not plausible for participants as they were asked to report the last two digits of their cell phone numbers, which ranged from 0 to 99.

However, anchoring effects for wine were significant at 1% level in the cathodal group, which suggested that decreased activities of right DLPFC increased anchoring effects, even if the anchors were not plausible. Comparing other two products, participants usually owned a common knowledge that the price of wine was expensive (e.g., more than 100), they were more likely to deem the anchors were implausible and irrelevant to the current task (Ravizza and Carter, 2008), and detect the conflicts between the rules for responding to cognitive sets trigged by anchors (Mansouri et al., 2007) and the appropriate cognitive sets (Ravizza and Carter, 2008). Therefore, participants were less susceptible to anchoring effects when the anchors were implausible. As DLPFC is engaged in the detection of conflicts of cognitive sets triggered by different contextual rules (Mansouri et al., 2007) and switching or shifting to the appropriate cognitive set (Ravizza and Carter, 2008), decreased activities of DLPFC in the cathodal group could weak the ability to detect conflicts of different cognitive sets and increased anchoring effects.

Meanwhile, although 30 s of stimulation did not produce significant neural altering effects on the cortex (Hummel et al., 2005; Willis et al., 2015), the placebo effect, which indicates the simple act of receiving any treatment (active or not) makes participants believe that it will work (De la Fuente-Fernández et al., 2001), may make difference of participants’ decisions between sham stimulation and pure behavioral experiments. Overall, we hold the idea that further investigation is needed to explain the conflicting results of existing studies.

When comparing effects of different tDCS conditions imposed on the behavioral biases, tDCS studies usually first build a measure or index of the behavioral biases, such as aggression (Riva et al., 2014), risk aversion (Ye et al., 2015), and trust (Wang et al., 2016), then perform statistical comparisons (e.g., *t*-test and ANOVA) for behavior of interest. However, there is not a direct measure of anchoring effects in the WTP paradigm (Ariely et al., 2003; Bergman et al., 2010; Fudenberg et al., 2012), which suggests a comprehensive consideration of multiple tests is needed to compare effects of tDCS conditions on anchoring effects. We mainly examined anchoring effects by investigating the significance of correlation and regression between WTP and anchors, which was an industry standard of existing studies (Ariely et al., 2003; Bergman et al., 2010; Fudenberg et al., 2012). We found robust results in both cathodal and anodal stimulation, which showed coefficients for all three goods were not significant in the anodal group and were all significant at 5% level in the cathodal group, while the coefficient in the sham group was significant at the 5% level for one item and at the 10% level for two of three items (see the results of the OLS model in **Table 5**). Meanwhile, paired *t*-tests in **Table 4** suggested that the level of anchoring effect in the cathodal group was more significant than those in the sham and anodal groups, while *post hoc* tests of ANCOVA showed the anodal group differed significantly from both the cathodal group (*p* = 0.002) and sham group (*p* = 0.04). These results suggested that the anodal stimulation decreased anchoring effects, while the cathodal stimulation increased anchoring effects.

We found that the activity of right DLPFC was related to anchoring effects. However, in a fMRI study, Tamir and Mitchell (2010) found that the activity of relatively dorsal aspects of the medial prefrontal cortex (MPFC), rather than the right DLPFC, played an important role in anchoring effects. Using whole brain parametric analyses, they identified a region in MPFC where activities were linearly correlated with self–other discrepancy when people used their own thoughts and feelings as an anchor for making inferences about others. Moreover, this anchoring effect was not correlated with activity in the right DLPFC.

The inconsistency between our study and that of Tamir and Mitchell (2010) can be explained because they used an experimental framework of social interaction involving mentalizing, the process in which participants inferred the mental states (e.g., thoughts or feelings) of others (Dufwenberg et al., 2011). Some studies have emphasized the role of increased activation of dorsomedial PFC (DMPFC) in the mentalizing processes (Tamir and Mitchell, 2010; Macoveanu et al., 2016). For example, Macoveanu et al. (2016) found frame conformity was associated with a significantly stronger activation of DMPFC than non-conformity. As both Tamir and Mitchell (2010) and Macoveanu et al. (2016)’s designs involve mentalizing in social interaction frameworks, we infer that the neural mechanisms of anchoring or framing may either involve mnemonic processes of cognitive sets which is related to DLPFC (Mansouri et al., 2007) or mentalizing processes which is related to DMPFC (Iacoboni et al., 2004; Tamir and Mitchell, 2010; Macoveanu et al., 2016), depending on the experiment design involves social interaction with others or not.

In this paper, we investigated the role of right DLPFC, rather than left DLPFC, in anchoring effects. In fact, many studies have suggested that the role of DLPFC in working memory is lateralized. The prior fMRI and positron emission tomography (PET) studies revealed that activation was predominantly left lateralized for verbal working memory and right lateralized for non-verbal and spatial working memory (Opitz et al., 2000; Reuter-Lorenz et al., 2000; Wager and Smith, 2003; Sandrini et al., 2008). Meanwhile, the selection of working memory items was associated with activation of the right DLPFC (Rowe et al., 2000; Rowe and Passingham, 2001). However, given the bilateral involvement of DLPFC on cognitive processes, it would also be important to test the roles of left DLPFC in anchoring effects. One direction is that we can induce bipolar balanced stimulation by placing tDCS electrodes bilaterally over the left and right DLPFC (i.e., left/right prefrontal hemispheric dominance). However, such a design cannot separate the specific contributions of the left and right DLPFC, and we still need follow-up studies to focus on the modulation of activity in one hemisphere alone (Sellaro et al., 2016). Thus, further studies on left DLPFC alone may help us have a deeper understanding of prefrontal hemispheric dominance in anchoring effects.

## Conclusion

The present research provides one of the first instances of evidence for the role of right DLPFC in anchoring effects. We found that tDCS induced modulation of cortical excitability, targeted at the right DLPFC, affected anchoring effects. Our results provide some supports for the psychological explanation that the standard anchoring paradigm can be seen a result of selective accessibility. These findings are important for those seeking to understand how anchoring effects are generated and human behavior is affected.

## Author Contributions

XY, JL, GW, and LQ designed experiment; JL, XY, and XL performed experiment; XY and DL analyzed data; XY and DL wrote the manuscript. The manuscript was approved by all authors for publication.

## Conflict of Interest Statement

The authors declare that the research was conducted in the absence of any commercial or financial relationships that could be construed as a potential conflict of interest.
